# Left to their own devices: Post-ELSI, ethical equipment and the International Genetically Engineered Machine (iGEM) Competition

**DOI:** 10.1057/biosoc.2013.13

**Published:** 2013-06-24

**Authors:** Andrew S Balmer, Kate J Bulpin

**Affiliations:** a Department of Sociology, University of Manchester, Arthur Lewis Building, Oxford Road, Manchester, M13 9PL, UK.; b Department of Sociological Studies, University of Sheffield, Elmfield Building, Northumberland Road, Sheffield, S10 2TU, UK. E-mail: sop09kjb@sheffield.ac.uk

**Keywords:** post-ELSI, iGEM, synthetic biology, human practices, collaboration, interdisciplinarity

## Abstract

In this article, we evaluate a novel method for post-ELSI (ethical, legal and social implications) collaboration, drawing on ‘human practices' (HP) to develop a form of reflexive ethical equipment that we termed ‘sociotechnical circuits'. We draw on a case study of working collaboratively in the International Genetically Engineered Machine Competition (iGEM) and relate this to the parts-based agenda of synthetic biology. We use qualitative methods to explore the experience of undergraduate students in the Competition, focussing on the 2010 University of Sheffield team. We examine how teams work collaboratively across disciplines to produce novel microorganisms. The Competition involves a HP component and we examine the way in which this has been narrowly defined within the ELSI framework. We argue that this is a much impoverished style of HP when compared with its original articulation as the development of ‘ethical equipment'. Inspired by this more theoretically rich HP framework, we explore the relations established between team members and how these were shaped by the norms, materials and practices of the Competition. We highlight the importance of care in the context of post-ELSI collaborations and report on the implications of our case study for such efforts and for the relation of the social sciences to the life sciences more generally.

## Introduction

This article is concerned with the issue of post-ELSI (ethical, legal and social implications) collaboration within the specific context of a rather heterogeneous collective of research themes increasingly termed ‘synthetic biology' (synbio or SB). Where the human genome project has become synonymous with the emergence and consolidation of the ELSI framework, emerging fields such as nanotechnology and SB are fast becoming entangled in what might be seen as a ‘post-ELSI' movement. In this article, we present our experiment with a post-ELSI form, content and method, within the context of SB and specifically with regard to the International Genetically Engineered Machine (iGEM) Competition. In particular, our work draws on, develops and responds to the ‘human practices' (HP) strand of post-ELSI research, constructed by Rabinow and Bennett (2009, 2012). We report on the creation of a novel form of reflexive ethical equipment, which we have termed ‘sociotechnical circuits'. We use the case of our engagement with the 2010 iGEM team from the University of Sheffield to explore some of the potential and pitfalls of post-ELSI collaboration with the life sciences. In this regard, our work forms part of the emerging discourse on the relationship of the social sciences to the natural sciences in the twenty-first century (Rose, 2013).

### Context: SB and the iGEM Competition

The common thread in the collective of actors working under the nomenclature of SB is an entanglement, both rhetorical and practical, of engineering and biology, which manifests in a diverse set of forms. O'Malley *et al* (2008) identify a range of themes, one of the more prominent being the ‘parts-based' approach that seeks to standardise genetic engineering materials and practices. The parts-based approach is particularly wedded to engineering logics and language, and explicitly adopts the notions of ‘devices', ‘parts', ‘standards', ‘circuits' and so on. According to this movement, the adoption of engineering logos promises to smooth the transformation of biological artefacts into industrial products by making them easier to control, predict and construct, essentially working to recreate the natural world according to the social norms of engineering (Calvert, 2010).

The iGEM Competition is one important sociotechnical form that has developed within the parts-based agenda. iGEM is intimately connected to parts-based work through its many contributions to the Registry of Standard Biological Parts (partsregistry.org/Main_Page) and its commitment to interdisciplinary teamwork. Indeed, Frow and Calvert (2013) argue that the main proponents of parts-based SB use iGEM as proof-of-principle for a broader research and policy agenda in the advancement of SB.

The Competition brings together students from across a range of disciplines to work collaboratively on the production of a ‘genetically engineered machine', usually a microorganism. The teams compete for medals (bronze, silver and gold) and for a limited number of trophies. Through these activities, the Competition aims to ‘foster scientific research and education' (igem.org/About) in SB. Since 2008, the Competition has included HP as part of its judging criteria and offers a specific award to the team contributing the ‘best human practices advance' (2011.igem.org/Judging). The manifestation of SB in iGEM thus makes for an excellent space in which to interrogate the potential contribution of social science to interdisciplinary collaboration and pedagogy and thus to the potential of post-ELSI work in the life sciences.

Social scientists have approached SB from a range of perspectives, with HP being the first to be specifically developed in relation to SB. HP, in its original form, is concerned with the diagnosis and creation of ethical equipment, ‘a practice situated between the traditional terms of method and technology' (Rabinow and Bennett, 2007a, p. 2) that can, among other applications, be used to think reflexively about the ethical dimensions of relationships enacted in SB and its governance. We report on our attempts to develop a reflexive device, partly inspired by this work on ethical equipment and other themes we develop later. We term our ethical equipment ‘sociotechnical circuits', a practical instantiation of reflexive practice adapted for use in the specific context of SB and the iGEM Competition.

**Figure 1:**
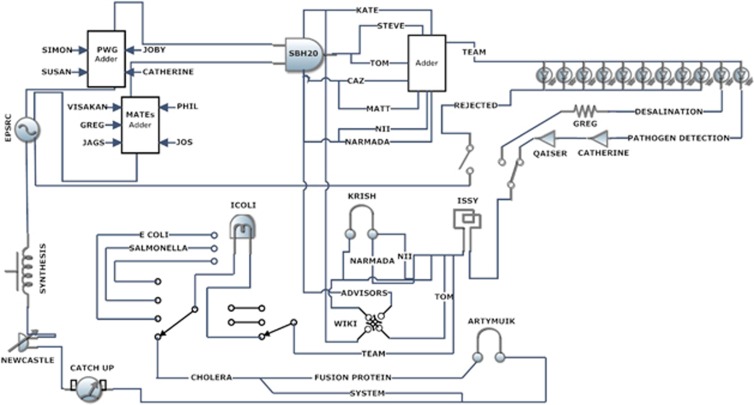
Sociotechnical circuit (Version 2).

The sociotechnical circuits are a way of representing the network of actors involved in an SB project that plays on the adoption of engineering logics and language within SB (see Figure 1). In short, they use the representational form of circuit diagrams as a way of mapping social and technical relations. They were developed through a number of empirical projects conducted collaboratively with the students that comprised the iGEM team based at the University of Sheffield in 2010. Our team had a specific goal by virtue of it being funded by an Engineering and Physical Sciences Research Council (EPSRC) Cross-Disciplinary Feasibility Account on SB, which required that the students come up with a project that related directly to problems faced by the water industry. As such, the team decided to create a ‘cholera biosensor' – an *E.coli* engineered to determine the presence of cholera in water. To win medals in the Competition, the teams must design new parts and use existing ones, which are catalogued in the Registry of Standard Biological Parts. iGEM has been the primary driver of the Registry as, year-by-year, it has expanded to include more teams from more countries producing more parts to populate it. As Campos (2012, p. 5) argues in relation to the development of iGEM: ‘Synthetic biology had gained a youthful and powerful new engine for ongoing part development – even as discussions about what exactly constituted a part continued apace'. Indeed, the iGEM Competition has been a vital element in the constitution of SB and in making the community and its ethos ‘stick' (Molyneux-Hodgson and Meyer, 2009). Although sociological studies have thus engaged with the emergence of SB, our current understanding of the experience of working with BioBrick parts, competing in iGEM and of how HP plays out in this context, is underdeveloped.

This article is based on qualitative data that comes from interviews, ethnographic observations and focus groups involving students and researchers participating in iGEM. We conducted semi-structured interviews with all 6 members of the Sheffield 2010 team, both during the Competition and afterwards (12 in total). In addition, we helped the team to themselves interview 13 other iGEM participants and 2 academic advisors from across 7 other institutions internationally (making 27 interviews total). Second, we engaged in detailed ethnographic observations of work inside and outside the laboratory over an intensive 10-month period, beginning from the advertising of the Competition at Sheffield, through picking a team, to choosing a project, implementing it and presenting it at MIT. This ethnographic work became collaborative as we developed the sociotechnical circuits and the team engaged in the reflexive work that it facilitated. The iGEM students devoted several hours each week to constructing the circuits and reflecting on the development of their project. Finally, we conducted a number of focus groups with the team, based on a series of topics at points throughout the project, and most significantly these included a lengthy discussion of the meaning of modelling and how the team felt about the process of working together.

In the following section, we show how HP in iGEM has been tied to broader movements in SB oriented towards industrialisation and interdisciplinarity. In later sections, we examine the origins of HP, relate it to other post-ELSI developments and report on its reconfiguration in iGEM through a more ELSI-style framing. We then introduce our innovation in ethical equipment, the ‘sociotechnical circuits' and describe their impact on the team's reflexive experience of the Competition. Finally, we reflect on this case study and its importance for SB more broadly, the potential and problems of a human practices approach to post-ELSI collaboration, and the wider implications for the relationship between the social sciences and the life sciences.

## iGEM, Interdisciplinarity and Industry

The desire to industrialise biotechnology is an important driver in the epistemological work of SB (see, for example, Calvert, 2012; Campos, 2012; Hilgartner, 2012) and forms a major element of its promissory capacity of bringing engineering into biotechnology. However, as Burggren *et al* (2010, p. 125) point out, the ‘relationship between biology and engineering is an old one. […] Yet the marriage between biology and engineering is neither easy nor automatic'. Clearly, work has to be done to make engineering and biology ‘fit'. For example, Mackenzie (2010, p. 181) argues that ‘biological work, techniques and materials are being re-configured under the rubric of design'. Indeed, the imagined future synthetic biologist would be, according to Ginkgo Bioworks,^1^ an entrepreneurial designer, ‘with far greater leverage than a traditional scientist working at the bench' (ginkgobioworks.com/tech.html). In effect, SB aims not only to reshape the material of biology, but also the life, imagination and practices of the scientist engaged in such work. The proposed reshaping of those lives is influenced by this particular industrial engineering ethos.

The iGEM Competition's values, goals and practices are similarly entangled with industrial engineering and entrepreneurial ambitions. Projects often have explicit industrial goals. Take, for example, the Washington team's winning project from 2011, which aimed to produce diesel from standardised parts engineered into *E.coli.* The Sheffield iGEM project was certainly in line with this general pattern, with its focus on producing parts and devices for the solution of intractable water industry problems.

In addition, the awards structure of the Competition involves ‘track prizes', some of which are organised along lines relating to major industries, for example, the ‘Best Food and Energy' project and there is an overall award for ‘Best Manufacturing Project'. Finally, and most recently in the story of iGEM, there has emerged a separate Competition and jamboree dedicated solely to industrial projects, titled ‘iGEM-Entrepreneurship' or ‘iGEM-E'. The iGEM-E aims to reward groups of students for developing business models for SB, to consider IP issues and work towards financial backing for their innovation (see: 2012e.igem.org/wiki/index.php/Main_Page; 2011.igem.org/wiki/index.php?title=Software/Team_Advancement&oldid=206106).

While the iGEM Competition is thus structured by many of the same values and ambitions as is SB more generally, it also comes with a more explicitly pedagogical aim. Students are not only tasked with producing sets of standardised parts to populate the Registry, but are also expected to embark upon a learning process that will take them beyond the confines of their parent discipline:

The competition format is highly motivating and fosters hands-on, interdisciplinary education. Biology students learn engineering approaches and tools to organize, model, and assemble complex systems, while engineering students are able to immerse themselves in applied molecular biology. (2010.igem.org/About)

The iGEM Website (igem.org/Start_A_Team) recommends that teams are formed with 8–12 students from various disciplines, backgrounds and levels of expertise, and the Sheffield advisors made this an important factor in selecting their team. The majority of the other teams in 2010 also had an interdisciplinary character, comprising students from computer science, physics, control engineering, molecular biology, genetics and so on. Although differing in their specificity, most seemed to have been constituted with some regard for the multiple expertise necessitated by the Competition's awards system, with prizes available for best model, part, characterisation etc.

One further aspect of this interdisciplinary character of the Competition has been the HP strand, which has facilitated the appearance of social scientists, designers, artists and a range of other actors within teams and as advisers to the teams. We now describe the import of HP within contemporary post-ELSI developments and investigate how it has been reconfigured in iGEM.

## iGEM, Post-ELSI and HP

The ELSI programme, which developed around the Human Genome Project, has now been firmly consolidated in scientific governance. Jasanoff (2004) argues that in becoming embedded in governance it has fostered a significant focus on the ethical issues associated with scientific applications, which comes at the expense of concern with the politics of scientific practice. The programme is entrenched in the Mode II knowledge economy, and therefore often functions to smoothen the transition of technoscience into public spaces and to maximise economic promise (Gibbons *et al*, 1994). As Nordmann and Rip (2009) point out, these factors produce a kind of speculative ethics concerned primarily with hypothetical futures.

Thus, attempts have been made to re-establish the reflexive potential of research and to reaffirm the political dimensions of oversight, accountability and governance functions. Novel modes have emerged, including constructive technology assessment (CTA) (Rip *et al*, 1995), real-time technology assessment (Guston and Sarewitz, 2002), upstream engagement (Wilsdon and Willis, 2004), midstream modulation (Fisher and Mahajan, 2006) and anticipatory governance (Barben *et al*, 2007). These forms of intervention have been principally concerned with ‘opening up' (Stirling, 2005) laboratory practices so as to enable scientists' to be more reflexive in their decision-making processes. In this regard, these practices seek to enhance scientists' understanding of the co-production (Jasanoff, 2004) of the technical and social in their everyday working lives and to re-emphasise politics in the practices of science over politics of the products. Particularly in the context of nanotechnology, these processes have influenced governance and sought to reposition social scientists upstream, embedding them within ongoing scientific work. Such attempts to produce more collaborative relationships between social scientists and natural scientists in the constitution of sociotechnical systems can be understood to form a ‘post-ELSI' movement.

These developments in nanotechnology and elsewhere have, in some contexts, set a precedent so that relationships between natural and social scientists are becoming normalised and formalised in funding arrangements, governance and everyday work. This has certainly been the case with SB. In the United Kingdom, for example, the 2008 Research Councils UK (RCUK) call for applications to establish SB networks highlighted the need to involve social science at an early stage (B.B.S.R.C., 2008, p. 5). What role social scientists would play in the networks was, however, unclear and became the subject of debate for an ESRC seminar series (www.genomicsnetwork.ac.uk/seminarseries), leading to a discussion document on how collaborations across the natural/social divide might be negotiated and developed (experimentalcollaborations.wordpress.com/). Calvert and Martin (2009) explored potential roles as ‘contributors' and ‘collaborators', highlighting the differences that are emerging in the extent to which social scientists become part of the work of sociotechnical production or remain distanced as more critical observers. Central to these debates over our position and the strategies for producing new forms of scientific practice have thus been a concern with finding ways to develop more reflexive practices in science with a view to understanding how knowledge production in social science and science might be configured differently.

Rabinow and Bennett (2009, p. 100), in their development of HP, argue that a post-ELSI approach must mean to work ‘alongside of and collaboratively with biologists and engineers'. The term HP emanates from Rabinow's^2^ group as part of the Synthetic Biology Engineering Research Centre (SynBERC). HP has thus emerged directly in relation to SB and specifically with regard to the parts-based movement as pursued at SynBERC. Rabinow and Bennett (2007b) propose that the social science contribution to SB should be in the design of contemporary ethical equipment.

Drawing on Foucault's (2001, p. 312) observation that ‘equipment is the medium through which logos are turned into ethos', the group attends to the ways in which the intellectual and material aims of the parts-based agenda demand that we rethink the equipment used by scientists, regulators, social scientists and so on to ethically respond to often unpredictable events in research, applications and the world more broadly. Rabinow (2003, p. 9) has argued for contemporary equipment that makes use of its ancient connection to care of the self and others, which ‘was not just a state of consciousness; it was an activity […] it was an essential dimension of a whole way of life [… and] was part of a broader pedagogy'. In this, he connects contemporary post-ELSI work on the processes of knowledge production to a longer tradition of attempts to negotiate the many and often conflicting demands placed on the self across all levels, from interpersonal relationships, through actions of the State, to the natural environment. Such an approach (Rabinow and Bennett, 2012, pp. 85–90, 152–154) thus links behaviour at the everyday individual level (for example, confidence, stubbornness, good faith and good intentions) to group dynamics and practices (the distribution of funding within a research group, the way in which expertise reinforces cooperation over collaboration) and to larger sequences of collective action (the contestation around ontologies of standards and parts and the regulation of dual-use in national and international governance).

Elsewhere, Rabinow and Bennett (2007b, p. 11) argue that the creation of equipment oriented to what they term ‘flourishing' would be central to the cultivation of forms of care of ourselves, others and the world, making flourishing both part of the practice and purpose of science, ethics and pedagogy. Flourishing, as they explain, ‘involves more than success in achieving projects; it extends to the kind of human being one is personally, vocationally, and communally, as well as the venues within which such human flourishing is facilitated and given form as practice' (Rabinow and Bennett, 2012, p. 9). Critically, then, HP can be partially distinguished from other post-ELSI endeavours in that its conceptualisation of practices emphasises a concern with the formation and care of self and how this relates to other levels of constitution. For our concerns, in this article, the orientation of HP to flourishing and care provides a conceptual framework within which to explore Calvert and Martin's (2009, p. 204) proposed ‘reciprocal reflexivity'. The use of such a theoretically rich analytical and pedagogical framework could be particularly timely, given the significance of the iGEM student Competition for the consolidation of the parts-based agenda of SB as a material, institutional and social reality (Rabinow and Bennett, 2012, pp. 141–142). In our work with the iGEM team, HP also had the benefit of being the term used in the Competition to designate the more socially oriented dimensions of the teams' projects. However, this complex and intellectually demanding theoretical disposition has struggled to find a foothold within iGEM or SB in its original form. Indeed, in the context of iGEM, the meaning and use of HP has become muddied by its recontextualisation within the Competition's norms and awards structure.

The first efforts to use HP analyses in an iGEM project came in 2007 with the work of Kristin Fuller, a social science undergraduate on the Berkeley team. Although Kristin kept a notebook including observations of the everyday practices in the lab, as well as on her own role and relation to the group as an embedded anthropologist, her work was ultimately instrumentalised to considering the intellectual property issues arising from the team's technical products (Rabinow and Bennett, 2012, p. 7). In this regard, the fledgling venture of HP into iGEM was constrained by the extant conditions of the Competition and norms of scientific work. Despite this, HP was institutionalised in 2008 as part of the awards system by the incorporation of a prize for the Best Human Practices Advance and became one important factor in teams trying to win a gold medal. It was introduced thus:

Issues? We've got issues! How will you sell your project if you have to give away the parts? What does your family think about your genetic engineering dreams? Will the world be a safe place if we make biology easy to engineer? How do the lessons of the past inform everybody's discussion going forward? Find a new way to help human civilization consider, guide, and address the impacts of ongoing advances in biotechnology. (2008.igem.org/Judging/Judging_Criteria)

The framing of HP here decontextualises the term from its post-ELSI origins in its relation to care and flourishing, and returns it to a determinedly object-oriented and public engagement-based framework. As such, HP projects have mainly constituted rather superficial addenda to the work of engineering novel bacteria, which focus mainly on the objects made by the teams rather than on the processes of making (Frow and Calvert, 2013). Consequently, this has led to a proliferation of design projects that have mostly sought to brand and package these promised technologies for public consumption. This fetishisation of the object is tied to the industrial rhetoric that permeates the iGEM materials and has further entrenched HP as an ELSI-style enquiry.^3^

This framing of ethics regularly came out in the interviews the Sheffield team conducted with other iGEM teams. Asking the interviewees what ethical issues they had encountered and whether they thought their project involved any ethical considerations, international iGEM participants almost universally replied that they had not encountered any ethical problems and did not expect to. They distanced their work from ethics by emphasising its modularity (‘our construct does not have any ethical implications' – European iGEM participant) and by displacing ethics to the future (‘It is nothing that we will encounter, it is like a future prospective. We will bear it in mind' – European iGEM participant). These ideas were often dependent on each other, for example, in the quote below:

European iGEM participant: Our team kept ethical issues in mind, but didn't really have it shape the project. You look at for example a team trying to clear up oil spills and they're facing more of an ethical aspect. Our main focus is the sciences and you keep the ethical aspects in mind.

In this regard, the practices of science were not seen as a territory requiring ethical consideration. Instead, ethical issues were often solely attached to the objects being considered. Although this articulation dominated, some participants did produce more nuanced accounts of the way in which ethics figured in SB – for example, by drawing attention to the vested career interests that synthetic biologists have in pushing the field forward. The participants made their most shrewd comments with regard to HP when they discussed ethics in relation to the challenge of completing the various requirements posed by the Competition:

European iGEM participant: If the project is [this specific application] then the ethics are not affecting the project and I guess if that part of the project is not successful then the ethics side of it is fairly irrelevant … there's no point in worrying about getting a gold medal [by doing some HP work] unless you've ticked all the boxes to get silver. So, all of the other bits of iGEM, if you want to actually get rewarded for it, rely on the biology working.

In this quote, we see again how ethics are understood as being separate from the empirical collaborative work of creating biological machines. However, we also see how this is partly because ethics is coded within the structures of iGEM, where considering ethical/HP issues is one important strategy for getting a gold medal. In this regard, the participant shows that the structure of the medal categories helps to constitute this separation of the laboratory aims from HP by making them sequentially significant. Since 2010, winning a bronze and silver medal are dependent on producing and characterising a BioBrick part or device respectively. To achieve a gold medal, teams must accomplish one or more of the following: include HP, help another iGEM team (for example, by characterising their part) or improve the functioning of an existing part in the Registry. The medals thus clearly enact the norms that serve the Registry, and in doing so they also enforce a system of value that locates HP both high up (in order to get a gold) and low down (all the technical aspects have to be solved first).

It is notable, however, that those teams awarded the HP prize in recent years have involved social scientists, and comprise work that moves somewhat closer to the post-ELSI agenda. In 2009, the prize for HP was shared between Paris and Imperial College London; in 2010, it went to Imperial again; and in 2011, it was also shared between Edinburgh and Arts–Science Bangalore. All of these teams have involved social scientists in one way or another, and this appears to have had some influence on the team's success in winning the award. However, the diverse nature of the winning teams' work also points to the broad interpretation of HP adopted in iGEM, which now regularly involves designers and artists. Moreover, as we discuss towards the end of the article, although these projects moved away from ELSI towards more inventive territory, they nonetheless retained more of a CTA-style attention to process in the service of object design, rather than adopting the HP-inclined focus on self-reflexivity and ethical equipment.

That the students interviewed by the Sheffield iGEM team largely report an ELSI-style interpretation of HP is thus in some tension with the style adopted by the HP winning teams, which evidences the current ambiguity around what should constitute an HP project. It is also related to how HP is rewarded via both the gold medal and the overall prize. Those teams winning the prize do seem to have benefited from the expertise and resources that come in collaboration with social scientists. This section points us towards the way in which power dynamics, resources, time, expertise and institutional histories play a role in the experience of iGEM. It alerts us to the possibility that, although HP has been largely reconceived within iGEM and is understood by most teams as an object-oriented, ELSI-style endeavour, its original conceptual framework may still be useful in understanding the experience of participation in iGEM, and that there is some hope for this style of enquiry in the Competition. In the remainder of the article, we mobilise ethical equipment and report on the development of our own form, specifically adapted for use in an iGEM project, and describe how this equipment helped stimulate a more reflexive approach to the Competition.

## Developing Sociotechnical Circuits

Although the challenge of taking HP seriously was great, the conditions were in our favour. Having been funded by an EPSRC grant, there was a small pot of money available to pay a team of talented undergraduates and to buy some materials. Importantly, the EPSRC grant involved a Co-Investigator, Susan Molyneux-Hodgson, from Sociological Studies who had appointed one of us, Andy Balmer, as a postdoc on the project with responsibility for guiding the team in their HP work. In addition, Kate Bulpin, a PhD student investigating education in the context of SB, was embedded as an ethnographer in the team and, as a team member helped them work on their HP. Susan had a long-standing, productive relationship with several of the science and engineering advisory teams. Andy was thus treated as an equal to the science and engineering advisers. This helped legitimate our presence in the project and contributed to the team's willingness to invest time, energy and self into the HP work.

We engaged in some informal conversations and more formal teaching around STS (Science and Technology Studies) and related themes, and discussed our previous work and current endeavours, meaning that the team was predisposed to venturing into somewhat unchartered waters with their HP efforts. Informed by the discussions we had around STS and HP, in particular the orientation to ‘socio' and ‘technical' interactions,^4^ the team decided that their HP work should aim to explore the various social and technical factors that came together in shaping the wider project. For example, the team examined how human and material forces had acted upon their choice of project topic and how team roles emerged and were consolidated.

We shared these questions in our work of observing and participating in the team; however, we also sought to evaluate the potential for ethical equipment within the specific context of the iGEM Competition and SB more broadly. Our roles and motivations were thus complex, as we were not only interested in producing good STS work and experimenting with post-ELSI forms, but also in helping the team accomplish their aims and in making the most of their experience. The emphasis in our iGEM HP project on the roles and relations of the team also served to sustain this ambiguity, as the students occasionally used these ideas to quiz us on our motivations and research. This begins to evidence how the collaboration proved useful in stimulating discussions that might otherwise have been impossible.

One important problem in fulfilling the promise of an equipmental version of HP was the issue of how to do HP in a short amount of time, with little training, scarce resources and a lack of instructive examples from previous Competitions. It struck us that these constraints were related to the kinds of problems that the programme of integrating engineering norms, such as standardisation, was intended to overcome in the context of SB. As such, in a reflexive twist on the relation between engineering and biology, between SB and HP, and thus on the language of devices (in SB) and equipment (in HP), we co-created (with the team) ‘sociotechnical circuits'. By inventing a version of ethical equipment that would help explore roles and relations, the work bridged the concerns of the team's project with those of our post-ELSI endeavours.

In short, the sociotechnical circuit adopts the language and symbolism of electrical engineering, much like SB, to describe the material and social constitution of the Sheffield 2010 iGEM project. However, it was not merely the visual representations of sociotechnical circuits that proved vital to our HP project; rather, it was the work of constructing the circuit diagrams that served as a reflexive practice and pedagogical tool that, within the constraints of the iGEM Competition, opened up potential avenues for care of the self and others. Before discussing the critical dimensions of the circuits as ethical, reflexive equipment, we now describe the practical method of creating the circuits.

**Figure 2:**
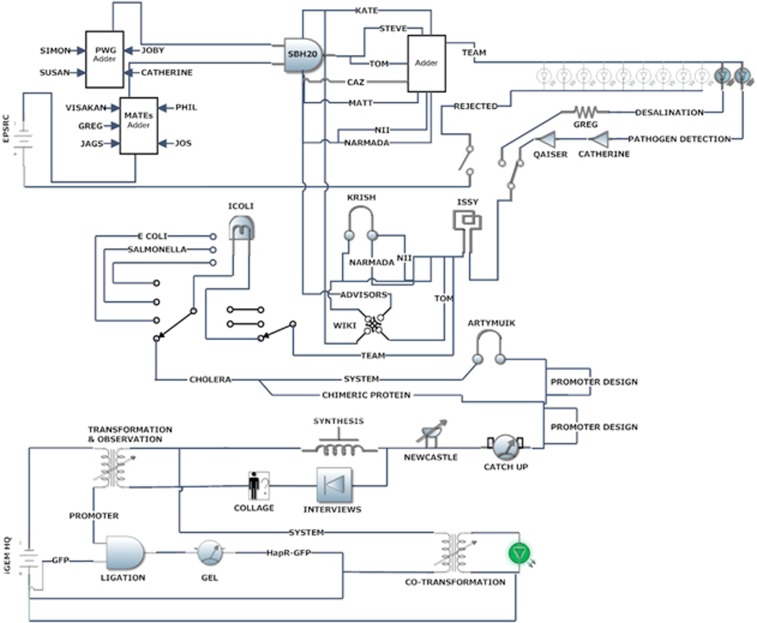
Sociotechnical circuit (Version 3).

**Figure 3:**
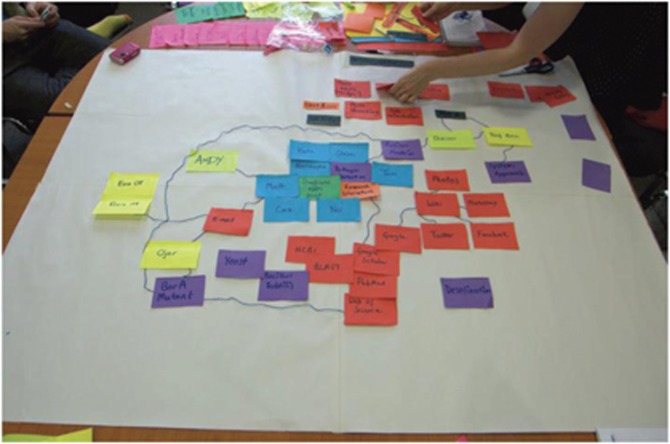
The team work with sticky notes and string to show relations between actors in the project during the first couple of weeks of activity.

Producing the circuits primarily involved mapping the various material and human ‘actors' involved in the project as it proceeded (Figures 1 and 2 show changes over time). Over the course of the project, we mapped activity using sticky notes,^5^ adopting different coloured notes for different ‘types' of actors (Figure 3). This marked the first level of standardisation and modularisation in the production of the circuits: actors, such as advisors to the team, were characterised according to their ‘function' – for example, one advisor was an ‘engineer', and another was a ‘microbiologist'. We took these sticky notes and arranged them in space according to their perceived conceptual, practical or physical relations. For example, the molecular biology advisor was placed next to the bioscience students, the molecular biology laboratory and the plasmid vector that he had assigned to them. These sticky note maps then served as the basis for producing a circuit diagram of the project's development, which involved representing each sticky note as a specific component from an electrical circuit. Decisions about the choice of component involved consideration of the kinds of ‘function' that those actors had played in the project's development. For example, one advisor who had played a role in discouraging a particular project idea was thus represented as a resistor; in contrast, another advisor was represented as an amplifier for the way in which they had helped consolidate the project idea that the team eventually pursued.

As representational forms, the circuit diagram and the images of sticky notes on which it was based failed to capture much of the complexity of the interactions between the various elements they depicted. In particular, the images did not adequately characterise change in these elements over time or their multiple functions and meanings in any given context at any given moment. Thus, using the above examples, individual advisors did not always constitute ‘amplifying' or ‘resisting' functions, and instead took on a diverse array of functions and roles over time and in any one particular situation. Rather than posing a problem for the circuits, it was this work of attempting to standardise and represent complex, interrelated phenomena and the problems that it caused that helped make the circuit diagram a reflexive tool. The circuit diagrams and maps of activity and actors constituted a practice for producing knowledge about the research project and how the students' and advisors' roles within the project were produced through these interrelations. In the following section, we describe the conceptual and methodological backdrop to this method of interacting with the students on the team.

## Circuit Diagrams as Ethical Equipment

It was not merely the representations themselves that formed the critical dimension of our HP work. Instead, the sociotechnical circuits represent an experiment with form, content and method that is particular to the demands of a post-ELSI approach. The circuits drew inspiration from several traditions and contemporary developments. First, from the tradition of new literary forms (Mulkay, 1984; Woolgar, 1988; Ashmore, 1989) that sought to give voice to the irony of STS work by reflexively engaging STS's own insights into the practices of science in its own presentation. Examples include the well-known ‘dialogues' staged by its practitioners who showed social science in the making. Second, we are responding to our science and engineering colleagues in the RCUK SB networks who regularly bemoaned the difficulty of understanding STS work, complaining about long texts stuffed with esoteric vocabulary. Such difficulties have led some scholars to experiment with form-giving and making, for example, from the lab of the ‘Anthropology of the Contemporary' (anthropos-lab.net/) or during the ESRC seminar series (www.genomicsnetwork.ac.uk/seminarseries/). Finally, the work of post-ELSI collaboration itself has been concerned with finding new ways to interact with natural scientists, and thus to develop twists on existing methods that facilitate these kinds of practices. In this regard, the circuits represent a new form, in the shape of the circuit diagrams themselves; a new reflexive method, in the collaborative production of those images; and new content, in their description of those novel collaborative practices.^6^ That the circuits operated in these three dimensions is key to understanding them as an experiment in reflexive ethical equipment.

Rabinow (2003) holds that one must understand such an equipment as a lifelong, practical activity of taking care in constituting the self. Such concerns are not unique to HP, as similar themes have also developed within STS, particularly in relation to the more post-structural feminist areas of the field. Drawing on this feminist trajectory, Puig de la Bellacasa (2011, p. 91) argues that:

One can make oneself concerned, but ‘to care' more strongly directs us to a notion of material doing. Understanding caring as something we do extends a vision of care as an ethically and politically charged *practice*, one that has been at the forefront of feminist concern with devalued labours.

Puig de la Bellacasa prompts us to tie care more closely to practices and the material–semiotic becoming of things. In this way, her notion of care is one that makes relations between human action and material things, and thus attends to the ways in which these matters of care *matter*. Both these theoretical positions emphasise the *work* of caring within local contexts and in relation to objects. Importantly, in combination, they alert us to the political charge of caring for those objects, practices and others that might otherwise be excluded and to the significance of care for the projects of education, of making the world and of solving problems.

Although we do not claim that our student colleagues developed this fundamental and long-lasting level of care of the self and others, or that they fully engaged on an intellectual level with this material, we do believe that they began to practice and understand some of the import of this conceptual framework through the circuits. The production of the sticky note maps throughout the project and their continual reconstruction and representation as circuit diagrams did engage students in some of the work of thinking through their relations and how they were connected to the material work in the lab and modelling. Under the following headings, we report on some of this caring and work of self-constitution in the day-to-day activities of the iGEM team.

### Roles of team members, fragmentation and care

In the process of building the circuits, conversation quickly turned to the roles that individual team members saw for themselves and others within the project. Reflection on how to standardise (using sticky notes or circuit symbols) the roles or ‘functions' of particular team members led to discussions of how early expectations regarding roles and activities in the team had shifted over the course of the project's successes and failures.

For example, as we discussed the emerging maps, one team member with a background in genetic engineering was identified as being the leader of the team. This implicit understanding had certainly contributed to the daily organisation of team behaviour, with the team leader often assigning activities and being the main point of contact for meetings with advisors. This identification of the leader, however, proved an ambivalent position for the individual in question, as it seemed to come not only with power over the direction of the project but also with the responsibility for its potential failure. Caught in this ambivalent position, he wrestled with continuing to take the lead while bearing the worry over problems that began to emerge and the expectations the advisors had for the team's success in Boston. In this respect, the circuits facilitated his self-examination, particularly in moments where important decisions had to be made, that involved him critiquing how his own actions, previous experiences and other factors outside of his control had helped contribute to his constitution as team leader.

When Rabinow (2003, p. 10) describes the Greek notion of care of the self, he reminds us that it involved an ‘unlearning of bad habits as well as the forming of good ones'. We cannot say that the team leader's self-examination was perpetual, but the HP work did encourage him to reflect more regularly on his position and habits. Indeed, when it came to designing the sequences for the parts, he began to make a more conscious effort to involve two other team members in this work, which he had otherwise been doing alone. This proved important in their self-constitution as the circuits began to show that they were integrally connected to the major genetic components in the circuits. As one of the pair reported: ‘it helped me feel more confident within the team, knowing that I had a valued purpose'. It also meant that more voices were heard in the design of the sequence, making manifest the changes in team dynamics within the material of the genetic ‘machine'.

In a similar way to the identification of the team leader, these biologically trained students were identified as having central roles in the daily activity of the laboratory. In producing the maps and circuit diagram, it became clear that they were both regularly identified in a particular space, surrounded (literally and representationally) by specific pieces of lab equipment, for example, the microwave, gel plates, scalpels, pipettes and so on. Over the course of the project, as time pressures became more significant, it became natural, when assigning the activities of the day, for them to conduct the procedures in which they had each become experts, as otherwise it would require them teaching someone else. This doubling up of human resources became unacceptable under the pressures of time and the demands of the Competition.

Such specialisation owing to time constraints, availability of resources, prior experience and so on also applied to other team members. For example, two students studying engineering degrees, who had familiarity with mathematical modelling, were quickly surrounded by a particular network of objects (the modelling software, scribbled equations, coffee machine, books and so on) and people who separated them from the activity of the laboratory. As one of them explained: ‘From the start I had the idea that I would take a main role in modelling but also get some experience in the lab. However, I quickly gave up on lab work after the first few weeks because the time frame for the project we had was not enough to learn the basics needed for the lab and apply them'.

**Figure 4:**
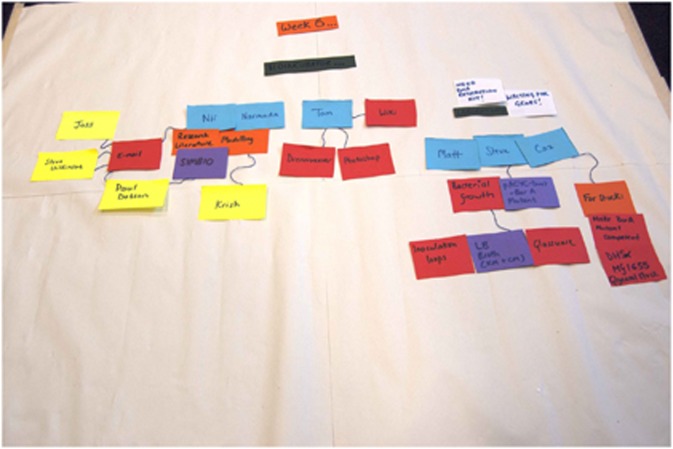
Sticky notes depict relations established in Week 6 of the project by which time the team had fragmented into smaller groups.

Owing to these contextual, material specificities of the laboratory and modelling work, the sub-teams were quickly separated by knowledge, time and space. Evidenced by the maps and diagrams (see, for example, Figure 4), the individual reflection on roles turned into a consideration of this fragmentation of the team along a disciplinary divide, with the engineers separated from the bioscientists. In interviews with other international teams, we found that many articulated a similar disjunction between the work of creating the system and of modelling it. This certainly plays out at the iGEM jamboree when most teams present their lab work and modelling work separately.

As we discussed the reasoning behind our team's separation, it quickly became clear that it was sensible to the team members that this be so. They discussed how they now possessed expertise in the lab and in the modelling that had taken on a certain value by virtue of the demands of the Competition's awards structure and the time pressures of the project. The work of producing the maps/circuits played a part in creating knowledge about the team and helped make it available in an immediate and sensible manner for the team members themselves. In an interview at the end of the project, one of the biologists reported that:

The circuits made good sense of how I'd arrived at various points on the project. In a similar way it showed how we'd all divided during the course of the project, indicating the point at which the lab/modelling divide occurred. Looking at it, [the] overall picture, the circuits do make sense of how my own work contributed to the final product.

In this regard, the maps and diagrams did not alter these divisions and instead ultimately served to reify the roles and positions that the students had come to inhabit. At first, this perhaps seems to be a ‘negative' outcome of the circuit diagrams, as the ethos of iGEM promotes a pedagogy of interdisciplinarity and promises that biologists will learn modelling and engineers will learn biology. However, iGEM is a time-pressured environment, and for teams to feel that they are responding appropriately to the Competition, this division of roles is a natural one. Indeed, other teams also seemed to understand this division of labour directly in relation to the Competition's rewards structure and argued that ‘you end up spending the most time on things you think the jury will value the most' (European iGEM participant).

As Rabinow (2003, pp. 9–10) argues the care of the self was ‘aimed at literally forming the subject […] care of the self passed through an elaborate network of relationships with others. The care of the self was highly social […] it was oriented from the self outward towards others, to things, to events, and then back to the self'. In this regard, the diagrams helped the students to articulate some of this network of relationships to others, things and events. Enrolling norms from the Competition, expectations of medals, the jury's decision making, the difficulty of learning lab techniques or modelling tools and the scarcity of resources the team members made sense of themselves and their work in a way that allowed them to acknowledge their separation without feeling that this was their explicit failing. Talk during these reflections highlighted the ways in which the team's experiences were thus constituted through networks of relationships that were not entirely within their control. This became more important as the Competition progressed and the team faced further engineering and social challenges.

### Norms, justice, Competition and care

The open-source style of the Registry implies a certain notion of democracy and equality in that each individual or team has the same access to the same information and is at their liberty to pick and choose which parts to use and alter. In the context of iGEM, this combines with the rhetoric (or myth) of meritocracy through which American universities largely organise themselves, in accordance with the belief that ‘individuals get ahead and earn rewards in direct proportion to their individual efforts and abilities' (Mcnamee and Miller, 2009, p. 2). Meritocracies suffer from a certain tendency to ignore background conditions, and thus assume (and reproduce) a kind of monoculture in which all individuals can be ranked according to the same criteria (Newfield, 2003, pp. 100–103).

In the development of the Competition at MIT, the progression to a form of meritocratic ideology was swift. Although in its first year, when the Competition comprised solely teams from MIT, many of them were awarded in one way or another, now, with a large international cohort, the teams compete around getting a medal or winning one of a limited number of trophies. The underlying logic in the Competition, which does not have any official mechanism for balancing out the teams in terms of their local conditions, is that talent will win through, that medals will be handed out justly and that the most deserving projects will get the trophies.

Calvert (2012, p. 174) has shown how ‘the parts-based approach aspires to develop different ways of owning and sharing biological systems, by attempting to transpose the normative values associated with open innovation regimes into the nascent synthetic biology community'. In this vein, one significant theme in the development of SB, the Registry and iGEM has been an increasing emphasis on characterisation as an important norm to enforce in the production and collection of parts. For example, this has gradually manifested in the iGEM judging criteria for the award of medals. The medal system was introduced in 2007 (007.igem.org/Jamboree/Awards), and in its first two years the requirements for the lowest award, a bronze medal, did not even include submitting a part to the registry and instead emphasised participation. By 2011 (2011.igem.org/Judging), the terms' ‘well characterised' and ‘outstanding documentation' had appeared in the bronze criteria.

We can see then that the iGEM Competition tries to carry the social and technical norms and values of parts-based engineering, democratic open source and meritocracy in material form from lab to lab, across the world, by posting out a uniform DNA distribution kit containing a selection of the parts from the Registry. In this respect, the parts and devices compel the teams to work towards the promised social dynamic. Frow and Calvert (2013) analyse this process as an attempt to enforce a new moral economy for biotechnology, but point out that biology continually resurfaces and reasserts itself. We agree that these materials are not sufficient in themselves to produce the practices required for a team to succeed in iGEM. Indeed, teams need a great deal of training and regular access to advisors not only with the requisite expertise in genetic engineering but also knowledge of the Registry and iGEM practices. They need funds to have parts synthesised, to purchase lab materials, to pay their way through the summer and travel to the regional (and possibly international) Competition. Moreover, they need time to learn, and thus they have to begin their education about SB well before the Competition officially starts. As such, the iGEM Competition and the teams that comprise it are currently caught between a certain rhetorical promise of global accessibility where talent wins through and where DNA assembly works just like clicking together Lego bricks and the less than perfect reality embodied in historical conditions and local constraints of time, resources and expertise that shape the worlds in which individual teams locally create their biological machines.

This tension became significant in the lives of our team. As they were confronted with the reality of getting the protocols and parts to work in their local context, they bemoaned their experience by reference to other large, well-funded, well-trained and closely advised teams, especially those in the United Kingdom and Europe, which served to sensitise our team to their own conditions and to the unfairness of the Competition. Survey results (Mitchell *et al*, 2011, p. 159) have suggested that some students and advisers have become concerned that the Competition is ‘growing stratified, with new or smaller teams unable to compete for prizes with established “powerhouse” teams'. Similarly, our team began to argue that no matter their ingenuity and dedication the sheer scale and cultural capital of these other teams would outweigh and outcompete them every time. They reached out to their advisers and to the resources at Sheffield more generally for support in this context.^7^

However, this was the first time that Sheffield University had really invested in iGEM. Moreover, none of the advisors had the requisite experience with iGEM protocols to be able to properly instruct the biologists in the protocols and norms required to work smoothly within the iGEM ethos. Indeed, the team's main molecular biology advisor did not want them to use the protocols or plasmids recommended by iGEM. Instead, he retained his own practices and trained them in using his plasmids and protocols, with the expectation that it would be relatively straightforward to recode this work into the standards required for parts to be submitted to the Registry. This meant that the team became more frustrated at the end of the project when having finally managed to get a glimmer of success with their engineered *E.coli*, they had to rework this into the iGEM standards and characterise it, following the demanding protocols set out on the wiki.

The team felt a little downtrodden at having managed only to accomplish a little of what they had hoped to have produced and – by the end of the project – did not have sufficient time to characterise their part and thus be worthy of a silver medal. It seemed to them that they had been climbing a steep hill where other teams had had the ground flattened for them by their advantage with resources and expertise. Needing to make sense of the injustice they felt and of how they now felt about themselves as young scholars with high expectations, they began to make use of the critical reflexive practices they had been developing through producing and reworking the circuits.

For example, members of the team began to distance themselves from the object they had produced by drawing attention to how it had always been decided for them that they would create a biosensor because the EPSRC grant had stipulated it. Indeed, although their choice was ostensibly open, as discussion progressed and ideas were developed, challenged and dispensed with, the biosensor was consolidated as the natural option. It was made clear that their object had to respond to challenges the industry faced and that it could not be too ambitious, partly because the water industry was presented as conservative.

The circuit diagrams had evidenced how one advisor acted as a ‘resistor' to many of the ideas, making it harder to pursue particular projects options, because his expertise had been used to mark these as undoable in the timeframe and because he had the most experience of traditional genetic engineering. Irrespective of the reality of this advice, it nonetheless had the effect of constraining the team's ideas so that as time moved on and it became imperative to choose a project, the most obvious choice had quietly become the biosensor. By the end of the lab work, then, as the team struggled to get their part characterised, they made reference to this experience of being channelled towards the biosensor to help explain their sense of distance from their work. This would have been much more difficult, had the team not spent significant amounts of time reflecting on how decisions had been made/constrained by human and material forces, and how the project had developed over time. The circuits thus helped them to take care of themselves in such a way that these various injustices regarding their freedom to choose and the discrepancies in resources did not too heavily have an impact on their self-esteem and sense of accomplishment.

## Discussion

As the results of the Competition were announced at the jamboree our team sat a little despondently, knowing that they were unlikely to get a silver medal, and as the slide appeared on the screen it was confirmed that we had got a bronze. We were pleased, nonetheless, as this was the first medal that Sheffield had received. However, our hopes for any further recognition as a success now lay with the HP work we had conducted. The team's presentation at the jamboree attracted a number of HP judges, as our wiki had marked us out as a potential contender for the award. However, in a question directed to the team following their presentation, one judge wished to understand how had our HP changed our project or improved our object? The team struggled to articulate this, as they had not used their HP to respond directly to their object but rather to their practices. This proved to be a decisive factor in the determination of the winner. Imperial College, London, had astutely tied their HP into an industrial design-style working cycle, showing how their project was shaped by these considerations and made much of the way in which their discussion with local experts in sociology had affected the imagined design of their envisaged product. This was praised in the ceremony as being a model for HP work and Imperial won the award.

The iGEM project was finished. It had been a long slog over a relatively short period of time. As we have shown, a number of factors contributed to shaping the project, constraining the team's ambitions and defining their experience of the Competition. Of these, time, industrialisation, expertise, resources, extant practices and the intransigence of the ELSI framework proved most significant. They were interrelated and were difficult to disentangle. As such, we present them consecutively in the following three discussion sections, but intertwine them with each other in our analysis of our case study of iGEM and its significance for SB more broadly, HP as a post-ELSI methodology and for post-ELSI collaboration with the life sciences.

### iGEM as a case in the study of SB

In the summer months, time was instrumental in ordering the sequences of action and shaping the priorities of the team. For professional synthetic biologists, the situation is more complex: they seem to have more time to produce their objects, but the pressures on the use of that time are far more potent. The experience of time in SB is importantly tied to the effort to industrialise genetic engineering work. This is a common theme in the contemporary life sciences, where traditional lab-bench biology is being reconfigured in line with industrial modes of production and sequencing of action (Jordan and Lynch, 1992; Vermeulen, 2009).

Moreover, our collaboration in iGEM has shown that the constraints of expertise and resources represent a significant barrier to developing HP work that tries to go beyond instrumental concerns. Our team succeeded in producing an excellent HP project but only with guidance from two advisors and with a team member with expertise in social science. Thus, they devoted time without much complaint to HP that might otherwise have gone towards laboratory and modelling work. This contrasts with Rabinow and Bennett's (2012) experience with professional colleagues. Partly this may be because of a power dynamic, in which our role was more authoritative than was theirs, where in SynBERC the power relation was the reverse, as the social science researchers were subject to a range of strategies for controlling their work. Although they had resources and ostensibly had authority, these were not sufficient to overcome the existing practices of controlling and allocating time.

Indeed, in our HP work, we discovered that time and resources played a critical role in cementing the division of labour between the lab and modelling teams. As each of these sub-teams spent more time developing their expertise in their respective areas and as the time remaining in the project ticked away, the possibility of our team members switching roles and engaging in a more interdisciplinary manner diminished. Our team responded to such time pressures by drawing on extant practices and resources, under the guidance of the existing expertise of their scientific advisers.

In professional SB contexts, this might contribute to the fact that extant practices of tinkering in genetic engineering are still very much in place within SB labs, as these actors continue to ‘kludge' at the nexus of existing and novel practices (O'Malley, 2009). SB actors seek to shift time and free up time for creative design by using the engineering ontology of parts and devices in order to produce greater efficiency in the production of novel organisms. However, as Shove (2004) and Southerton (2003) have convincingly shown in everyday life, the drive to save time and produce greater efficiency has the ironic effect of helping to constitute the experience of ‘harriedness'. Similarly, professional researchers' experiences of time are increasingly harried (Garforth and Červinková, 2009), particularly by virtue of the inculcation of political and corporate mechanisms for evaluating and governing science (for example, work packages, Gant charts, deliverables). As such, synthetic biologists may find that the genetic materials they wish to transform are more compliant than are lab practices and governance. Industrialisation produces a time regime that turns increased efficiency into a demand for even more production and labour. Therefore, efforts to free up time in this context of changing practices may conversely increase the experience of harriedness.

### The potential and problems of a post-ELSI HP approach

The exposition of HP has received criticism from some STS scholars concerned to examine what exactly it adds to extant STS theories and mechanisms. As Edmond and Mercer (2009) contend, HP is unlikely to integrate substantively with the everyday work of SB, as such efforts must be carried out within a broader institutional and governmental framework that may well be resistant to prolonged and demanding reflexive work. Moreover, they argue that in focussing on the ‘good life' and flourishing, HP appears to promote self-regulation, perhaps naively imagining that scientists will transform themselves and their practices when there is no institutional or financial imperative to do so.

Contrary to this, we think that Rabinow and Bennett (2012) do propose some possible imperatives to sociotechnical transformations of the relations between the natural and social sciences in their assessment of the increasing pressures posed by global problems such as climate change or the regulatory challenges of dual use. However, we agree that these are not yet understood by scientific actors as tied to their everyday practices. Rabinow and Bennett have also more recently acknowledged the difficulty of getting HP to work in SynBERC in their analysis of the breakdown of those relationships and do so in a manner that makes sense of the existing norms of science and politics. As they argue (Rabinow and Bennett, 2012, p. 153), extant practices of lab safety and governance of genetic materials emphasise the dangerous ‘Other' as an imagined security threat, often in the guise of the rogue scientist or garage biologist. The corollary of this is that reflection on the scientific self is unnecessary, as ‘good' scientists, with good intentions, using the correct procedures are not a risk. In this regard, investments of time and resources in the work of HP are seen as a waste. The obstinacy of these downstream, object-oriented governance frameworks in science and engineering are also reflected in the predominant forms of HP work at iGEM. Although the more ‘upstream' post-ELSI approaches to iGEM HP invite reflection on the sociotechnical construction of technologies, the focus remains on the product over enquiry into everyday scientific practice. Indeed, HP has not taken hold in iGEM and SB or in STS circles. Most obviously, this might be because the language and conceptual framework of HP is challenging at best and obscure at worst. In addition, although clearly connected to a particular tradition of anthropology of science, the notion of ethical equipment does not provide a transparent guide to its application as a method for social research. As such, working with HP takes time, intellectual resources and emotional and academic labour in its application. In this regard, we social scientists are subject to similar time regimes in which the application of extant STS concepts and methods promises a more efficient route into knowledge production and its representation. Finally, the public breakdown of the SynBERC collaboration and some of the subsequent animosity is perhaps a poisoned chalice for researchers interested in adopting post-ELSI frameworks.

Most significantly, with regard to the critique of HP ethos, we feel that flourishing is a rather privileged term – not all people can flourish to the same degree as there are inequalities in the distribution of resources that might allow scientists to flourish. As such, HP might too easily connect to the established logic of meritocracy and serve to reinscribe inequalities rather than alleviate them. Our adoption of Puig de la Bellacasa's feminist notion of care into the programme of HP is thus an important refinement of care in the ethos of flourishing as it encourages care for neglected labours, individuals and relations.

### Implications for post-ELSI collaboration with the life sciences

As we have already begun to argue, the constraints on the life sciences (as we have shown with iGEM and SB) are important in shaping the dynamics of possible collaborative relationships. Not least, those factors of time and the obstinacy of extant practices. Moreover, those same structures shape social research and constrain *our* ability to experiment with novel methods and forms. HP is useful in this regard, as it calls for a reflexive imagination that can take its own activities into account within its application. Recent calls for ‘artful' (Back and Puwar, 2012), ‘playful' (Balmer, 2013) and ‘experimental' (Balmer *et al*, 2012; Rabinow and Bennett, 2012) approaches to methods and the curation of sociological knowledge thus must not only be sensitive to these constraints but might also benefit from making them visible in their application.

In this regard, inventive post-ELSI mechanisms such as the development of ethical equipment must ask for a pause in the sequencing of collaborations. This can be a difficult request. The drivers of industrialisation in the life sciences and the influence of political mechanisms of bureaucracy are potent barriers to more reflexively engaged scientific practices. As Menzies and Newson (2007) report, scientists already bemoan that there has been a loss of time for reflection on oneself and for more dialogical connections with colleagues. As such, scientists must already negotiate ‘time to think' together (Garforth and Červinková, 2009, p. 171) in the form of lab meetings, coffee breaks and so on. Social scientists and natural scientists seeking to engage must thus work together to actively produce reflexive moments as part of the ethical equipment we invent. It is in these moments in which the work of collaboration can be undertaken, examined and renegotiated. Arguing for a pause is thus a political act in itself, and one we have to understand as a form of resistance to extant pressures shared across the natural and social sciences. In this, we may have at least one common point of everyday friction with which to join with our natural science colleagues in shifting research practices.

Developments in SB that build mechanisms of convenience and time saving may come at the expense of care. As we have argued, care is vital to our attempts to understand and change sociotechnical relations. The work of caring for collaborations and challenging established modes of governance of the life sciences can be understood as a form of devalued labour. However, there are opportunities to evidence its value, as we began to see in our HP work with the iGEM team. For example, our sociotechnical circuits did help the team to identify the roles individuals were taking, to understand their consolidation, to react positively to the constraints they faced at Sheffield and to contextualise their ostensible failure to do better within the injustices of unequal resource distribution. Of course, this might not be terribly important to those lab groups that benefit from these inequalities.

These seem to us to be important lessons to be learned in caring for oneself and others not least in iGEM, but also in SB and perhaps in the life sciences more generally. Inequality of resources does not only affect small teams of undergraduates, but also large teams of research professionals working in an economic climate that is ever more stringent and competitive. Moreover, the work of our colleagues in SB and the life sciences is often framed within a promissory language of solving international problems, many of which are related to inequalities of, for example, access to health care, clean water or food. HP and cognate post-ELSI approaches are thus a more cogent framework from which to assess the relations between laboratory practices and the problems they seek to address than is the more object-oriented ELSI approach. The attention that HP gives to the everyday experience of scientists, to care of ourselves, collaborations and the world makes for a vital addition to existing post-ELSI frameworks.

Our work in developing and implementing the sociotechnical circuits shows that novel forms and methods can be productive for both parties involved in reciprocal reflexive collaboration (Calvert and Martin, 2009). Our ethical equipment more directly involves our science colleagues in the production of knowledge about knowledge production. For example, in producing the circuits the team often contested their own representation, negotiated with each other over their understandings of the project and were able to engage reflexively with what the circuits told them about their experience. This was not only important for them but also deeply stimulating for us as ethnographers of science, as we formed part of this work of contestation and renegotiation. As such, we more actively took part in the shaping of the team's interrelations and in the production of knowledge than we might have without this method of engagement. Post-ELSI approaches that attend to care of self and others are thus a promising direction for mutually productive collaboration between social and natural scientists.

## Conclusion

In this article, we have explored the potential of HP as a post-ELSI methodology by use of the iGEM Competition as a case study in SB. Our work contributes towards understanding of the experience of participating in the emerging interdisciplinary field of SB and evidences the complex demands placed on both novices and professionals. It suggests that these constraints are similarly important in shaping the possibility of collaboration across the natural and social sciences in this context and in the life sciences more generally. We have explored the potential of an experiment with form, content and method in the shape of the sociotechnical circuits, which are designed to think through some of these challenges and to test out a novel collaborative, reflexive approach. The circuits and the lessons learned from them for SB, post-ELSI and our relation to the life sciences are promising, but they also highlight the durability of existing frameworks in both scientific and social scientific work, not least ELSI, industrialisation and the governance of academic life. Therefore, we must continue to invent new forms, experiment with new collaborative methods, insist on reflexive moments and remain resilient in the face of failure, if we are ever to succeed in more fully collaborating.

## Footnotes

1 One of the more prominent companies advocating a parts-based SB framework.

2 Recent developments in SynBERC's organisational structure and ethos have meant that Rabinow has left the Centre. The HP thrust has been rather tellingly re-named as ‘practices' and is now led by Drew Endy, a synthetic biologist. We recognise the relevance of these changes for HP, but feel that proper consideration of this issue is outside the scope of this article.

3 However, there have been other more critically informed design projects that have engaged substantively with the process of design, the socio-economic conditions of doing SB, the unpredictability of technology's life course and the human factors to be considered in constructing technical solutions.

4 Which we examined through a cursory chat about Actor Network Theory and other STS themes interested in such entanglements.

5 See the following link for a selection of images that show us developing the maps of action using sticky notes during one session: picasaweb.google.com/117594342876677821323/ SociotechnicalCircuits#5532510456214100194.

6 Collaborative relations were examined both between the engineers and biological scientists in the team, and our relationship with those two groups.

7 The judges are aware of this difference in cultural capital, resources and so on between teams and sometimes explicitly favour the smaller teams, for example, in choosing Cambridge over the juggernaut of Heidelberg in 2009 (Jane Calvert, 2013, personal communication). Nonetheless, the inequalities remain, and larger teams are at a distinct advantage in completing the various requirements of obtaining a gold medal.
